# Teneligliptin Decreases Uric Acid Levels by Reducing Xanthine Dehydrogenase Expression in White Adipose Tissue of Male Wistar Rats

**DOI:** 10.1155/2016/3201534

**Published:** 2016-08-29

**Authors:** Chihiro Moriya, Hiroaki Satoh

**Affiliations:** Department of Diabetology, Endocrinology, and Metabolism, Fukushima Medical University, 1 Hikarigaoka, Fukushima, Fukushima 960-1295, Japan

## Abstract

We investigated the effects of teneligliptin on uric acid metabolism in male Wistar rats and 3T3-L1 adipocytes. The rats were fed with a normal chow diet (NCD) or a 60% high-fat diet (HFD) with or without teneligliptin for 4 weeks. The plasma uric acid level was not significantly different between the control and teneligliptin groups under the NCD condition. However, the plasma uric acid level was significantly decreased in the HFD-fed teneligliptin treated rats compared to the HFD-fed control rats. The expression levels of xanthine dehydrogenase (*Xdh*) mRNA in liver and epididymal adipose tissue of NCD-fed rats were not altered by teneligliptin treatment. On the other hand,* Xdh* expression was reduced significantly in the epididymal adipose tissue of the HFD-fed teneligliptin treated rats compared with that of HFD-fed control rats, whereas* Xdh *expression in liver did not change significantly in either group. Furthermore, teneligliptin significantly decreased* Xdh* expression in 3T3-L1 adipocytes. DPP-4 treatment significantly increased* Xdh* expression in 3T3-L1 adipocytes. With DPP-4 pretreatment, teneligliptin significantly decreased* Xdh* mRNA expression compared to the DPP-4-treated 3T3-L1 adipocytes. In conclusion, our studies suggest that teneligliptin reduces uric acid levels by suppressing* Xdh* expression in epididymal adipose tissue of obese subjects.

## 1. Introduction

The prevalence of type 2 diabetes mellitus has increased dramatically worldwide, mainly because of changes in lifestyle, such as decreasing exercise and increasing high-fat diets. Obesity is the hallmark of the metabolic syndrome and represents a major global health problem that is frequently associated with the development of chronic diseases, including type 2 diabetes mellitus and cardiovascular disease [1]. A complex interorgan cross talk scenario between adipose tissue and other central and peripheral organs underlies the progression of these diseases, with adipose tissue on top of the cross talk hierarchy [2].

Use of dipeptidyl peptidase-4 (DPP-4) inhibitors is a strategy for glucose-lowering treatment in type 2 diabetic patients [3]. DPP-4 inhibitors were first approved for clinical use in 2006 with the DPP-4 inhibitor sitagliptin, and, thereafter, many other DPP-4 inhibitors have been introduced into clinical practice [4]. Teneligliptin, one of the DPP-4 inhibitors, has a unique structure characterized by five consecutive rings, which produce a potent and long-lasting effect [5, 6]. The gut-derived glucagon-like peptide-1 (GLP-1) plays important roles in both postprandial and long-term glucose homeostasis by increasing glucose-stimulated insulin secretion and inhibiting glucagon secretion [7]. DPP-4 is an enzyme that rapidly degrades circulating GLP-1, and, therefore, DPP-4 inhibitors prevent the inactivation of GLP-1 and, consequently, increase the circulating active GLP-1 levels above physiological levels that have antidiabetic actions [3]. In addition, DPP-4 is a ubiquitously expressed transmembrane glycoprotein that cleaves N-terminal dipeptides from a variety of substrates, including growth factors and hormones, neuropeptides, and chemokines, such as incretin hormones [8]. The expression of DPP-4 is substantially dysregulated in a variety of disease states, including inflammation, cancer, obesity, and diabetes [9]. It has also been reported that DPP-4 released from adipose tissue is positively correlated with an increasing risk score for the metabolic syndrome. DPP-4 release is strongly correlated with adipocyte size, potentially representing an important source of DPP-4 in obesity. Therefore, it has been suggested that DPP-4 may be involved in linking adipose tissue and the metabolic syndrome [10].

Recently, it has been reported that adipose tissue produces and secretes uric acid through xanthine oxidoreductase (XOR) and that its production is enhanced in obesity [11]. Uric acid is also one of the risk factors for cardiovascular diseases [11, 12]. In mammals, XOR can exist in two enzymatic forms: xanthine dehydrogenase (XDH) and xanthine oxidase (XO). XO induces oxidative stress in the process of uric acid production. On the other hand, cardiac insufficiency and obesity produce a hypoxic state that leads to oxidative stress, which activates XO. Oxidative stress is highly relevant to aging and the development of various aging-related cardiovascular diseases and insulin resistance. Thus, inhibition of XO suppresses the oxidative stress of uric acid, which improves vascular endothelial dysfunction, heart failure, and insulin resistance [12].

We hypothesized that teneligliptin might have pleotropic effects in these tissues. DPP-4 inhibitors can improve glycemic control by prolonging the effect of GLP-1. Many studies have already reported that teneligliptin improves not only blood glucose but also the lipid profile and early-phase insulin secretion [13–17].

In the current study, the effect of teneligliptin on uric acid metabolism was examined in male Wistar rats. It was found that teneligliptin decreased uric acid levels in high-fat diet- (HFD-) fed rats, but not normal chow diet- (NCD-) fed rats.

## 2. Methods

### 2.1. Materials

Teneligliptin was donated by Mitsubishi Tanabe Pharma Corporation (Osaka, Japan). 3T3-L1 preadipocytes were purchased from American Type Cell Collection (Manassas, VA, USA). Male Wistar rats were obtained from Charles River Laboratory Japan, Inc. (Kanagawa, Japan). The high-fat diet (HFD) (60% w/w, #D12492) was purchased from Research Diet Inc. (New Brunswick, NJ, USA). Isoflurane was purchased from Intervet K.K. (Tokyo, Japan). Pentobarbital was purchased from Kyoritsu Pharmaceutical Co. (Tokyo, Japan). DMEM, streptomycin, trypsin, fetal bovine serum (FBS), and TRIzol reagent were from Invitrogen Life Technologies (Carlsbad, CA, USA). The RNeasy kit was obtained from QIAGEN Inc. (Valencia, CA, USA). The iScript cDNA Synthesis Kit and the iQ SYBR Green Supermix were from Bio-Rad Laboratories (Richmond, CA, USA). DPP-4 Activity Fluorometric Assay Kit was purchased from BioVision Incorporated (Milpitas, CA, USA). Uric Acid Assay Kit was purchased from Cell Biolabs Incorporated (San Diego, CA, USA). All other reagents were purchased from Sigma (St. Louis, MO, USA).

### 2.2. Animal Studies

Six-week-old male Wistar rats (Charles River Laboratory Japan, Inc.) were housed individually under controlled 12-hour light, 12-hour dark cycles and temperature conditions (25°C) and had free access to water and a NCD or 60% HFD (Research Diet, Inc.). The male Wistar rats were fed with NCD or HFD with or without teneligliptin (~4.0 mg/kg body weight/day) for 4 weeks. The rats received a fresh diet every 3 days, and food consumption rates and body weight gains were monitored every 3 days. After the indicated diet for 4 weeks, the rats were fasted for 6 hours and then anesthetized in an induction chamber with 3% isoflurane. Plasma samples were obtained in the presence of EDTA-Na from the aorta of rats, and they were then promptly euthanized with pentobarbital (180 mg/kg body weight). The epididymal adipose tissue and liver samples were dissected and immediately frozen in liquid nitrogen and stored at −80°C for subsequent analysis. All procedures were performed in accordance with the Guide for Care and Use of Laboratory Animals of the NIH and were approved by the Animal Subjects Committee of Fukushima Medical University, Japan.

### 2.3. Uric Acid Level Measurement

Plasma uric acid levels were analyzed by uricase POD methods in a private laboratory (SRL Laboratory, Tokyo, Japan) [18].

### 2.4. Cell Culture and Cell Treatment

Mouse 3T3-L1 cells were cultured and differentiated as described previously [19, 20]. Unless otherwise indicated, adipocytes were used 14 days after differentiation. After incubation in serum-free DMEM high glucose for 3 hours, differentiated 3T3-L1 adipocytes were treated with the indicated concentration of teneligliptin and/or 200 ng/mL DPP-4 for 1, 3, 6, or 12 hours before each assay.

### 2.5. Quantitative Real-Time Reverse-Transcription PCR Analysis

Total RNA samples were extracted from cells, epididymal adipose tissue, and liver samples with TRIzol reagent, and total RNA was further purified using the RNeasy kit with RNase-free DNase I treatment according to the manufacturer's instructions. Total RNA (1 *μ*g) was reverse-transcribed with the iScript cDNA Synthesis Kit according to the manufacturer's instructions (Bio-Rad Laboratories, Inc.). Quantitative real-time PCR was performed with a Bio-Rad system using iQ SYBR Green Supermix and specific primer pairs (Table 1) selected with Primer Express software (Applied Biosystems). The relative mass of specific RNAs was calculated by the comparative cycle of threshold detection method according to the manufacturer's instructions.

### 2.6. DPP-4 Activity Measurement

DPP-4 activity in tissue homogenates and plasma sample was determined using a DPP-4 Activity Fluorometric Assay Kit (BioVision Incorporated) according to the manufacturer's instructions. Fluorescence intensity (excitation/emission = 360/460 nm) was measured every 10 minutes by Varioskan Flash 2.4 (Thermo Fisher Scientific K.K.).

### 2.7. Uric Acid Secretion Measurement

Uric acid in cultured medium of differentiated 3T3-L1 adipocytes with 10 *μ*M teneligliptin and/or 200 ng/mL DPP-4 for 24 hours was determined using a Uric Acid Assay Kit (Cell Biolabs Incorporated) according to the manufacturer's instructions. Fluorescence intensity (excitation/emission = 530/590 nm) was measured by Varioskan Flash 2.4 (Thermo Fisher Scientific K.K.).

### 2.8. Statistical Analysis

Data are presented as means ± SEM. Statistical significance was tested with repeated measures analysis of variance (ANOVA). Statistical significance was defined as *P* < 0.05.

## 3. Results

### 3.1. Overall Animal Characteristics

The effect of teneligliptin was examined in both NCD-fed and HFD-fed male Wistar rats.  Table 2 shows some of the general characteristics of the teneligliptin and control groups at 4 weeks. In the NCD-fed rats, body weight, average daily food consumption, and fasting plasma glucose levels were not significantly different between the teneligliptin and the control groups. Compared with the control group, HFD feeding led to a significant increase in body weight but with no significant change in fasting plasma glucose levels. In the HFD-fed rats, body weight, average daily food consumption, and fasting plasma glucose levels were also not significantly different between the two groups.

### 3.2. Effects of Teneligliptin on Uric Acid Metabolism in Male Wistar Rats

**Figure 1:**
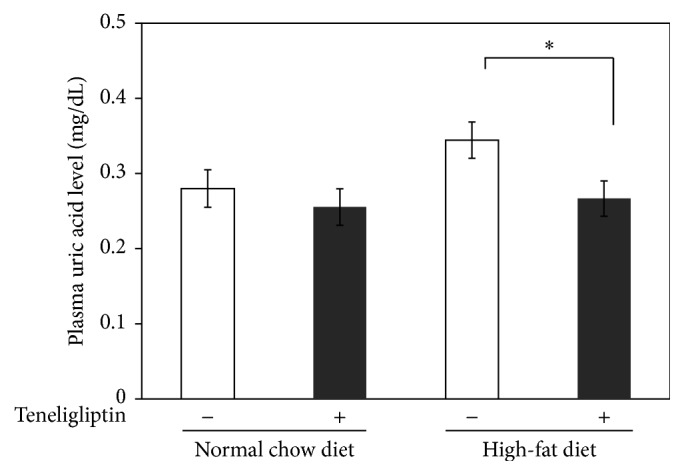
Effects of teneligliptin on plasma uric acid levels in male Wistar rats. The rats were fed with NCD or HFD with or without teneligliptin (~4.0 mg/kg body weight/day) for 4 weeks. After 4 weeks, the rats were fasted for 6 hours, and then plasma samples were obtained in the presence of EDTA-Na from the aorta of rats under anesthesia. The plasma uric acid levels were measured by a private laboratory (□, control group, NCD-fed rats: *n* = 10, HFD-fed rats: *n* = 9; ■, teneligliptin group, NCD-fed rats: *n* = 9, HFD-fed rats: *n* = 9). Data are the means ± SEM. ^*∗*^ *P* < 0.05 versus the control rats.

In the NCD-fed rats, plasma uric acid levels were not significantly different between the teneligliptin and the control groups (Figure 1). Interestingly, in the HFD-fed rats, plasma uric acid levels were significantly decreased by 21% (from 0.34 ± 0.02 mg/dL to 0.27 ± 0.02 mg/dL; *P* < 0.05) in the teneligliptin group compared with the control group (Figure 1).

### 3.3. Effect of Teneligliptin on the Uric Acid Synthesis Gene (*Xdh* mRNA) in Liver and Epididymal Adipose Tissues of Male Wistar Rats

**Figure 2:**
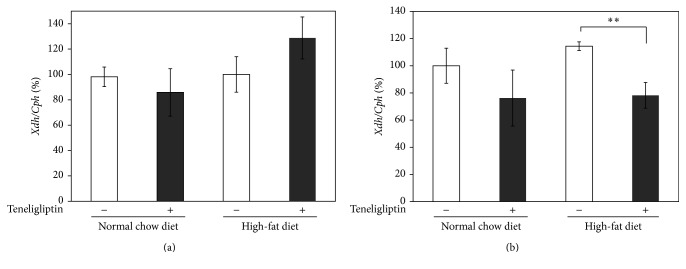
Effects of teneligliptin on xanthine dehydrogenase (Xdh) in liver (a) and epididymal adipose tissues (b) of male Wistar rats. Total RNAs extracted from liver tissue and epididymal adipose tissue of the control group (□: NCD-fed rats: *n* = 10, HFD-fed rats: *n* = 9) and the teneligliptin group (■: NCD-fed rats: *n* = 9, HFD-fed rats: *n* = 9) were used for gene expression analysis of Xdh. Levels of Cph were used for normalization of sample loading. Data are the means ± SEM. Data are expressed relative to NCD-fed control values. ^*∗∗*^ *P* < 0.01 versus control rats.

To explore the potential cellular mechanisms involved in the teneligliptin-induced decrease of plasma uric acid levels, quantitative real-time PCR analysis was performed on total RNA from epididymal adipose tissue and a liver sample in selected chow-fed rats. The expression level of xanthine dehydrogenase (*Xdh*), which is one of the key enzymes in the uric acid synthesis pathway, was measured. The expression of* Xdh* in liver tissue was not significantly altered by teneligliptin treatment under either NCD or HFD conditions (Figure 2(a)). On the other hand, the expression of* Xdh* in epididymal adipose tissue was reduced significantly by 32% (*P* < 0.01) in the HFD-fed teneligliptin treated rats compared to the HFD-fed control rats, whereas the expression of* Xdh* in epididymal adipose tissue did not change significantly in the NCD-fed control rats and the NCD-fed teneligliptin treated rats.

### 3.4. Effect of Teneligliptin on DPP-4 Activity in Plasma, Liver, and Epididymal Adipose Tissues of Male Wistar Rats

**Figure 3:**
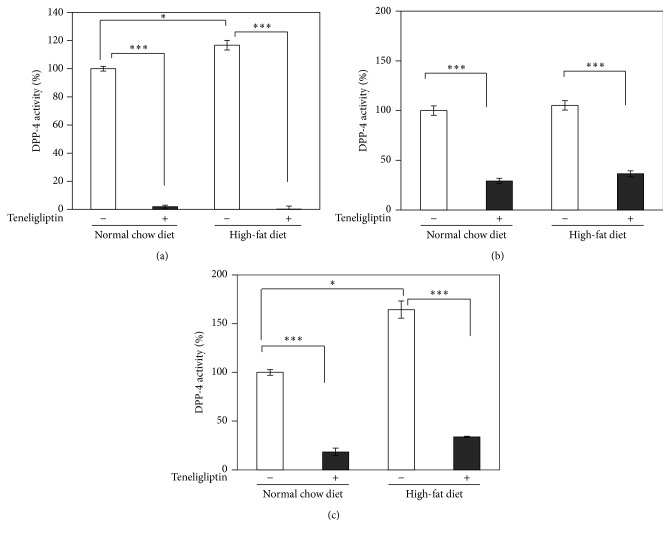
Effects of teneligliptin on DPP-4 activity in plasma (a), liver (b), and epididymal adipose tissues (c) of male Wistar rats. The activity levels of DPP-4 from plasma, liver tissue, and epididymal adipose tissue of the control group (open squares: NCD-fed rats: *n* = 3, HFD-fed rats: *n* = 3) and the teneligliptin group (closed squares: NCD-fed rats: *n* = 4, HFD-fed rats: *n* = 4) were measured by DPP-4 Activity Fluorometric Assay Kit (BioVision Incorporated). Data are the means ± SEM. ^*∗*^ *P* < 0.05 versus control rats; ^*∗∗∗*^ *P* < 0.001 versus control rats.

To evaluate the efficacy of teneligliptin in plasma, liver, and epididymal adipose tissues of male Wistar rats, the activity levels of DPP-4 were measured. The plasma DPP-4 activity was decreased significantly by more than 95% (*P* < 0.001) in both the NCD-fed and the HFD-fed teneligliptin groups compared to the NCD-fed and the HFD-fed control groups, respectively (Figure 3(a)). Moreover, the plasma DPP-4 activity was increased significantly by 17% (*P* < 0.05) in the HFD-fed control rats compared to the NCD-fed control rats (Figure 3(a)). The activity of DPP-4 in liver reduced significantly by 71% (*P* < 0.001) and 65% (*P* < 0.001) in the NCD-fed and the HFD-fed teneligliptin groups compared to the NCD-fed and the HFD-fed control groups, respectively (Figure 3(b)). On the other hand, the activity of DPP-4 in epididymal adipose tissues reduced significantly by 82% (*P* < 0.001) and 79% (*P* < 0.001) in the NCD-fed and the HFD-fed teneligliptin groups compared to the NCD-fed and the HFD-fed control groups, respectively (Figure 3(c)). Moreover, the activity of DPP-4 in epididymal adipose tissue was increased significantly by 64% (*P* < 0.05) in the HFD-fed control rats compared to the NCD-fed control rats (Figure 3(c)), whereas the activity of DPP-4 in liver did not change significantly between the NCD-fed and the HFD-fed control rats (Figure 3(b)).

### 3.5. Effects of Teneligliptin on Uric Acid Synthesis Gene (*Xdh*) in 3T3-L1 Adipocytes

**Figure 4:**
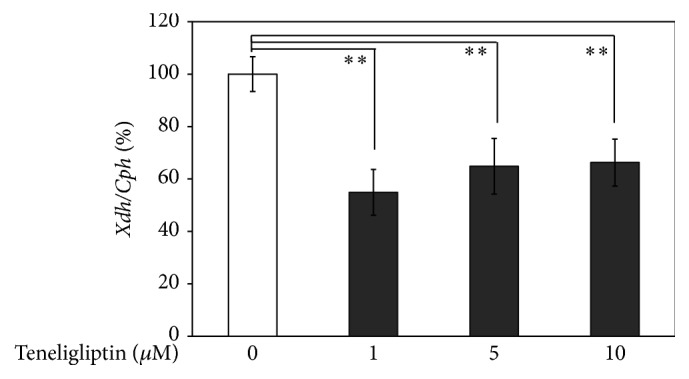
Effects of teneligliptin on* Xdh* expression in 3T3-L1 adipocytes. Serum-starved 3T3-L1 adipocytes were treated without (□) or with teneligliptin (■; 1, 5, and 10 *μ*M) for 3 hours. Total RNAs extracted from all cells were used for gene expression analysis of Xdh. Levels of Cph were used for normalization of sample loading. Data are the means ± SEM of 3 independent experiments (1 experiment performed with 6 samples). Data are expressed relative to control values. ^*∗∗*^ *P* < 0.01 versus control cells.

**Figure 5:**
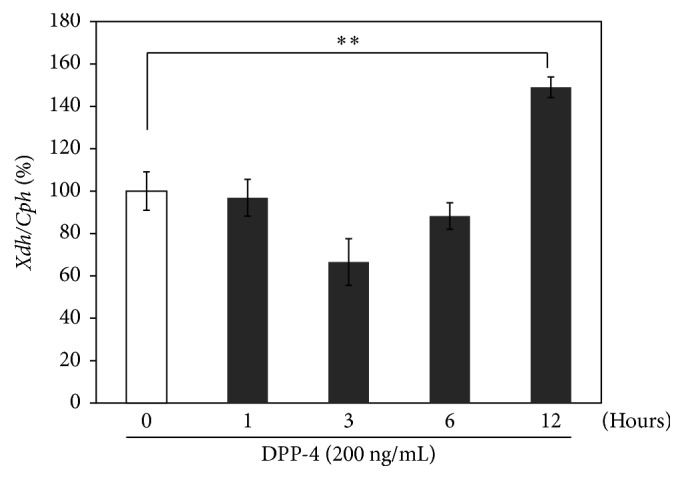
Effects of DPP-4 on* Xdh* expression in 3T3-L1 adipocytes. Serum-starved 3T3-L1 adipocytes were treated without (□) or with DPP-4 (■; 200 ng/mL) for the indicated periods (1, 3, 6, and 12 hours). Total RNAs extracted from all cells were used for gene expression analysis of Xdh. Levels of Cph were used for normalization of sample loading. Data are the means ± SEM of 3 independent experiments (1 experiment performed with 6 samples). Data are expressed relative to control values. ^*∗∗*^ *P* < 0.01 versus control cells.

**Figure 6:**
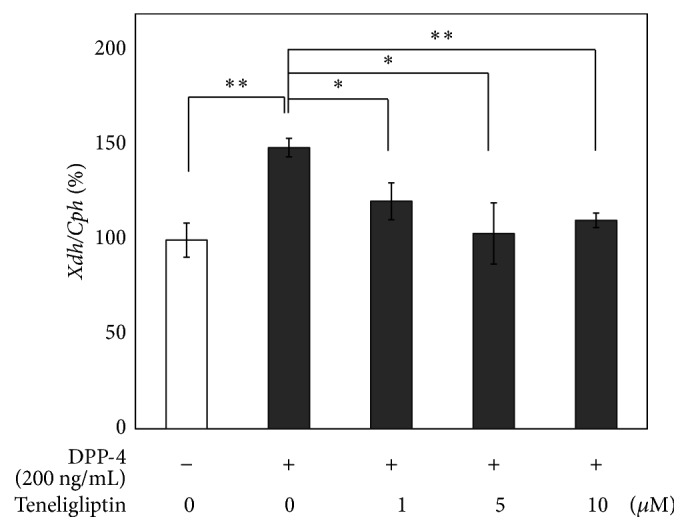
Effects of DPP-4 and teneligliptin on* Xdh* expression in 3T3-L1 adipocytes. Serum-starved 3T3-L1 adipocytes were treated without (□) or with DPP-4 (■; 200 ng/mL) and teneligliptin (1, 5, and 10 *μ*M) for 12 hours. Total RNAs extracted from all cells were used for gene expression analysis of Xdh. Levels of Cph were used for normalization of sample loading. Data are the means ± SEM of 3 independent experiments (1 experiment performed with 6 samples). Data are expressed relative to control values. ^*∗*^ *P* < 0.05; ^*∗∗*^ *P* < 0.01 versus DPP-4 treated control cells.

Next, whether treatment with teneligliptin altered the expression of* Xdh* in 3T3-L1 adipocytes was investigated. Quantitative real-time RT-PCR was performed on total RNA from serum-starved 3T3-L1 adipocytes that were treated with or without teneligliptin (1, 5, and 10 *μ*M) for 3 hours. As shown in Figure 4, teneligliptin significantly decreased the expression of* Xdh* by 45% (*P* < 0.01), 35% (*P* < 0.01), and 34% (*P* < 0.01) at 1, 5, and 10 *μ*M concentrations, respectively, in 3T3-L1 adipocytes. Moreover, as shown in Figure 5, treatment for 12 hours with DPP-4 (200 ng/mL), a novel adipocytokine that impairs insulin sensitivity in an autocrine and paracrine fashion [10], significantly increased the expression of* Xdh* by 49% (*P* < 0.01), whereas DPP-4 treatment for 6 hours did not significantly change the expression of* Xdh*. With DPP-4 (200 ng/mL) pretreatment for 12 hours, teneligliptin significantly decreased the expression of* Xdh* by 19% (*P* < 0.05), 30% (*P* < 0.05), and 26% (*P* < 0.01) at 1, 5, and 10 *μ*M concentrations, respectively, compared to the DPP-4-treated 3T3-L1 adipocytes (Figure 6).

### 3.6. Effects of Teneligliptin and DPP-4 on Uric Acid Secretion from 3T3-L1 Adipocytes

**Figure 7:**
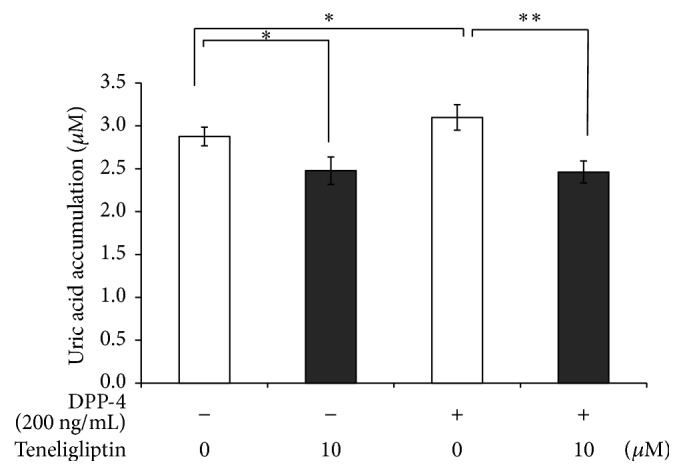
Effects of teneligliptin and DPP-4 on uric acid secretion from 3T3-L1 adipocytes. Serum-starved 3T3-L1 adipocytes were treated without (□) or with teneligliptin (■; 10 *μ*M) and/or DPP-4 (200 ng/mL). Uric acid secretion for 24 hours of incubation in the presence of the indicated concentration of teneligliptin or DPP-4, respectively. Data are the means ± SEM of 3 independent experiments (1 experiment performed with 4 samples). ^*∗*^ *P* < 0.05; ^*∗∗*^ *P* < 0.01 versus the value in the absence of teneligliptin.

Finally, whether treatment with teneligliptin and DPP-4 altered the secretion of uric acid from 3T3-L1 adipocytes was investigated. Serum-starved 3T3-L1 adipocytes were treated without or with teneligliptin (10 *μ*M) and/or DPP-4 (200 ng/mL). We examined uric acid secretion for 24 hours in the presence of the indicated concentration of teneligliptin or DPP-4. As shown in Figure 7, the presence of DPP-4 (200 ng/mL) significantly increased the accumulation of uric acid from 3T3-L1 adipocytes by 8% (*P* < 0.05) compared to the control. Furthermore, addition of teneligliptin (10 *μ*M) significantly reduced the rates of both spontaneous and DPP-4 stimulated uric acid accumulation from 3T3-L1 adipocytes by 13% (*P* < 0.05) and 21% (*P* < 0.01) (Figure 7). The protein content of the cells was not significantly changed by treatment with teneligliptin and DPP-4.

## 4. Discussion

Teneligliptin, a novel, highly selective DPP-4 inhibitor, is an antidiabetic drug that improves glycemic control without causing weight gain or increasing hypoglycemic risk in patients with type 2 diabetes mellitus. Although the glycemic efficacy of teneligliptin is well known, the nonglycemic efficacy and mechanisms of teneligliptin are not well understood. In this study, under the HFD condition, but not under the NCD condition, teneligliptin decreased plasma uric acid levels by reducing* Xdh* expression in adipose tissue but not liver. In addition, in 3T3-L1 adipocytes, DPP-4 increased* Xdh* expression, and teneligliptin decreased DPP-4-induced* Xdh* expression. These findings raised the possibility that teneligliptin may decrease plasma uric acid levels by inhibiting DPP-4 activity in adipose tissue.

DPP-4 is ubiquitously expressed on numerous different cell types, including epithelial cells, fibroblasts, and leukocyte subsets. Mechanisms that regulate DPP-4 gene transcription and enzymatic activity are not fully understood. It has recently been reported that adipocytes released DPP-4 in a differentiation-dependent manner [10]. In addition, DPP-4 expression in adipose tissue was increased in obese compared to lean subjects, a fact that is reflected by the increased release of DPP-4 from adipose tissue explants of obese subjects compared with lean subjects [10]. Circulating DPP-4 concentrations were increased in obese subjects and correlated with parameters of the metabolic syndrome, such as body mass index, waist circumference, and plasma fasting insulin concentration [10]. Furthermore, DPP-4 exerted autocrine and paracrine effects and impaired insulin signaling [10]. Thus, DPP-4 is a novel adipocytokine and biomarker, and it is a potential link between obesity and the metabolic syndrome.

A central finding in the present study is that teneligliptin, one of the DPP-4 inhibitors, reduced plasma uric acid levels in HFD-fed rats by downregulation of* Xdh* expression in adipose tissue. This observation is strongly associated with the upregulation of DPP-4 expression and release in adipose tissue of obese subjects. Therefore, DPP-4 increases* Xdh* expression and teneligliptin decreases DPP-4-induced* Xdh* expression in 3T3-L1 adipocytes. DPP-4 stimulates* Xdh* expression, and then* Xdh* expression promotes the production of uric acid.

Hyperuricemia is a key risk factor for the development of gout, renal dysfunction, hypertension, dyslipidemia, diabetes, and obesity. Hyperuricemia occurs as a result of the increased uric acid production, impaired renal uric acid excretion, or a combination of both [21]. Endogenous production of uric acid is mainly from the liver, intestines, and other tissues such as muscles, kidney, and vascular endothelium [22]. Recently, it has been reported that adipose tissue produces and secretes uric acid through XOR and that its production is enhanced in obesity [11]. In mammals, XOR can exist in two enzymatic forms: XDH and XO. The present results suggest that inhibition of* Xdh* expression in adipose tissue is important in the treatment of diabetes. Another study also suggested that knockdown of XOR promoted transcription of a PPAR*γ* [23]. Oxidative stress associated with XO production through the process of uric acid damages fat cells and vascular endothelial cells. In addition, in 3T3-L1 adipocytes, uric acid increased monocyte chemotactic protein (MCP-1) gene expression, which induced macrophages and inflammation, and decreased the expression of adiponectin. In addition, adding an antioxidant agent suppresses this reaction [24]. Aggravation of insulin resistance by such cell inflammation would exacerbate sugar metabolism itself. In other words, hyperuricemia can be related to multiple risk factors for atherosclerosis caused by the fat tissue. Suppressing the uric acid level is very important in diabetes therapy, because the goal of diabetes treatment may be to reduce the complications.

In this study, the effects of uric acid inhibition in the adipose tissue are caused by inhibiting DPP-4; therefore, we supposed that these effects are DPP-4 inhibitors common effects. We have presumed that one of the reasons is that linagliptin suppresses XOR activity in 3T3L-1 adipocytes [25]. Although the report did not take into account the uric acid production amount in adipose tissue in the methods of XOR activity measuring, it could be interpreted as the amount of uric acid in all over the tissue was reduced by linagliptin. This is consistent with the results of the present study. The effect that teneligliptin has reduced the uric acid is considered class effect. We have assumed this effect does not depend on each DPP-4's inhibition of own binding differences or its binding pocket. Sitagliptin is grouped into class 3 the same as teneligliptin, although sitagliptin increased the plasma uric acid level in humans [26]. Moreover, sitagliptin and linagliptin are the same nonpeptide bonds. Therefore, we suppose that similar effects may occur by performing the cell experiments using other DPP-4 inhibitors. Nevertheless, in animal experiments, we consider that the effect does not appear in other DPP-4 inhibitors, because of the differential transferability to adipose tissue. The amount of distribution of the other DPP-4 inhibitors in adipose tissue is very low compared to that of teneligliptin in 24 hours after drug administration. It is not detected in linagliptin, vildagliptin, and alogliptin after 24 hours [27–29]. In sitagliptin, drug concentration in adipose tissue was much lower than that of teneligliptin [30, 31]. Furthermore, in the present study, it was 12 hours later that the* Xdh* expression increased to its highest level after pretreatment with DPP-4 in 3T-3L1 adipocytes. Therefore, it is also important to maintain plasma concentrations of DPP-4 inhibitors. Teneligliptin can maintain plasma concentrations because of its terminal elimination half-life of 26.9 hours [5].

On the other hand, whereas the inhibition of DPP-4 activity by teneligliptin has been confirmed in liver and in adipose tissue of male Wister rats under both HFD and NCD conditions,* Xdh* mRNA expression in liver was not significantly changed. Although we do not know the precise mechanisms underlying these differences in experimental results in liver and adipose tissue, the possibility exists. (1) The liver is the organ that highly expresses DPP-4 compared to adipose tissue [32]. (2) The inhibition effect of DPP-4 activity by teneligliptin in liver attenuates less than those in adipose tissue. Therefore, we presume that the inhibition effect of* Xdh* expression by teneligliptin might differ according to the inhibition effect of DPP-4 activity.

However, we have presumed that there are at least two limitations in this study as follows: (1) The standard methods for the measurement of XOR and XO activity have not been established yet. The current method for the measurement of XOR or XO evaluates the amount of uric acid production in the sample tissue by adding xanthine. However, this current method cannot measure accurately XOR or XO activity because the abundant uric acid is contained in any tissue [33]. Therefore, we have measured the expression of* Xdh* mRNA in this study, even though the measurement of* Xdh* mRNA expression has the possibility to evaluate the inadequate uric acid metabolism. (2) Although the uric acid metabolism differs between humans and rat, we found that teneligliptin decreased the uric acid level since teneligliptin decreased the* Xdh* mRNA expression in adipose tissue of the HFD-fed rats and 3T3-L1 adipocytes.

In conclusion, the present data suggest that teneligliptin reduces uric acid levels by suppressing* Xdh* expression in epididymal adipose tissue of obese subjects. Therefore, teneligliptin is more effective for controlling blood glucose and hyperuricemia in patients with type 2 diabetes mellitus.

## Figures and Tables

**Table 1:** Primers used for real-time RT-PCR.

Target sequence	Primers	Primer sequence	Band size (bp)
Mouse Xdh MN_011723	mXdh-2819-F	5′-GGATGCTAATCGCAGAATAC-3′	120
	mXdh-2938-R	5′-GCTTCTGGTTGAAGTGAGTC-3′	

Rat Xdh NM_017154	rXdh-2579-F	5′-GAAGACTGGGACTGTAGTGG-3′	124
	rXdh-2702-R	5′-GGGGATCTTATAGGCGTTAT-3′	

Cyclophilin A (Cph) BC106030	Cph-96-F	5′-CTCCTTTGAGCTGTTTGCAG-3′	325
	Cph-420-R	5′-CACCACATGCTTGCCAT-3′	

**Table 2:** Body weight, food intake, and fasting plasma glucose measurements in NCD-fed and HFD-fed rats.

	Normal chow diet		High-fat diet	
	Control	Teneligliptin	Control	Teneligliptin
Number	10	9	9	9
BW (g)	367.6 ± 6.9	355.8 ± 16.7	415.0 ± 10.3^*∗*^	432.3 ± 9.3^*∗*^
Food intake (g/day)	24.9 ± 0.6	24.4 ± 0.7	20.3 ± 0.3	20.6 ± 0.3
FPG (mg/dL)	158.0 ± 9.1	161.6 ± 9.1	163.0 ± 3.8	162.6 ± 2.8

Means ± SE.

BW, body weight; FPG, fasting plasma glucose.

^*∗*^ *P* < 0.05 versus the normal chaw diet control rats.

## Abbreviations

DPP-4: Dipeptidyl peptidase-4
GLP-1: Glucagon-like peptide-1
XOR: Xanthine oxidoreductase
XO: Xanthine oxidase
Xdh: Xanthine dehydrogenase
HFD: High-fat diet
NCD: Normal chow diet.
